# All Trans-Retinoic Acids Facilitate the Remodeling of 2D and 3D Cultured Human Conjunctival Fibroblasts

**DOI:** 10.3390/bioengineering9090463

**Published:** 2022-09-11

**Authors:** Yuri Tsugeno, Tatsuya Sato, Megumi Watanabe, Megumi Higashide, Masato Furuhashi, Araya Umetsu, Soma Suzuki, Yosuke Ida, Fumihito Hikage, Hiroshi Ohguro

**Affiliations:** 1 Departments of Ophthalmology, School of Medicine, Sapporo Medical University, Sapporo 060-8556, Japan; 2 Departments of Cardiovascular, Renal and Metabolic Medicine, Sapporo Medical University, Sapporo 060-8556, Japan; 3 Departments of Cellular Physiology and Signal Transduction, Sapporo Medical University, Sapporo 060-8556, Japan

**Keywords:** three-dimensional spheroid cultures, human conjunctival fibroblast (HconF), all-trans-retinoic acid (ATRA), TGF-β2

## Abstract

Vitamin A derivative, all-trans-retinoic acid (ATRA), is known to be a potent regulator of the growth and differentiation of various types of cells. In the present study, the unidentified effects of ATRA on superficial and vertical spreading conjunctival scarring were examined. The study involved the use of two-dimensional (2D) and three-dimensional (3D) cultures of human conjunctival fibroblast (HconF) cells in the presence or absence of TGF-β2. The effects of ATRA (1 μM) on superficial or vertical spreading conjunctival scarring were evaluated by the barrier function by trans-endothelial electrical resistance (TEER) and FITC dextran permeability measurements and real-time metabolic analysis, as well as the physical properties, namely, the size and stiffness, of 3D spheroids, respectively. In addition, the expressions of several related molecules, including extracellular matrix (ECM) molecules, ECM modulators including a tissue inhibitor of metalloproteinases (TIMPs), matrix metalloproteinases (MMPs), and ER stress-related factors, were examined. ATRA significantly induced (1) an increase in TEER values and a decrease in FITC dextran permeability, respectively, in the 2D monolayers, and (2) relatively and substantially increased the size and stiffness, respectively, of the 3D spheroids. These ATRA-induced effects were further enhanced in the TGF-β2-treated cells, whereas the TGF-β2-induced enhancement in glycolytic capacity was canceled by the presence of ATRA. Consistent with these physical and morphological effects, the mRNA expressions of several molecules were significantly but differently induced between 2D and 3D cultures by ATRA, although the presence of TGF-β2 did not substantially affect these gene expression levels. The findings reported in this study indicate that ATRA may exacerbate both superficial and vertical conjunctival fibrosis spreading independently of TGF-β2-induced changes.

## 1. Introduction

Conjunctival scarring is a well-known and common complication of several ocular surface-related diseases, including infections, traumatic injuries, and others in the wound healing response after ocular surgery [1]. Such conjunctival wound healing responses can be characterized by two different mechanisms, that is, (1) superficial changes such as re-epithelialization and wound contraction and (2) vertical changes in the form of subconjunctival fibrous scar formation [1]. Within these mechanisms, transforming growth factor-β (TGF-β) is recognized as a critical causative factor [2,3].

A derivative of vitamin A, all-trans-retinoic acid (ATRA), is a potent regulator of the growth and differentiation of various types of cells [4,5]. Pharmacologically, ATRA possesses anti-inflammatory action by the suppression of nuclear factor–κB (NF-κB) signaling [6], as well as exerting an antifibrotic effect by attenuating the action of TGF-β action [7]. In fact, it has been reported that a derivative of ATRA inhibited TGF-β–induced liver fibrosis by suppressing the expression of the collagen *1A2* gene [8], and an isomer of ATRA, 9-Cis-retinoic acid, has been reported to attenuate the TGF-β–induced fibrotic changes within the cultured human mesangial cells [7]. In addition, such ATRA-induced anti-TGF-β effects have also been considered to be therapeutic candidates for modulating TGF-β subconjunctival wound healing, which is often observed post-surgery [9,10]. However, our knowledge of the drug-induced effects of ATRA toward two different responses, superficial and vertical conjunctival scarring formation as above, is currently limited. In our recent study, we successfully established in vitro models of replicated superficial and vertical scarring formation using a three-dimension (3D) spheroid culture, in addition to the conventional two-dimension (2D) cell culture of human conjunctival fibroblasts (HconF) cells [11]. This prompted us to examine the drug-induced effects of ATRA with respect to these established models.

Here, to study the drug-induced effects of ATRA on these 2D and 3D cultured HconF cells, we performed the following measurements: (1) barrier functions of the TGF-β2 treated 2D cultured HconF monolayers by trans-endothelial electron resistance (TEER) and FITC dextran permeability, (2) real-time mitochondrial and glycolytic cellular functions, (3) measurements of the size and hardness of the TGF-β2-treated 3D HconF cell spheroids, and (4) qPCR and/or immunocytochemistry of major extracellular matrix (ECM) molecules, their modulators, and endoplasmic reticulum (ER) stress-related factors.

## 2. Materials and Methods

### 2.1. Preparations of the 2D and 3D Cultured Human Conjunctival Fibroblasts (HconF)

Two-dimensional cultures of HconF cells (ScienCell Research Laboratories, Carlsbad, CA, USA) were cultured in 150 mm 2D culture dishes at 37 °C in Fibroblast Medium (FM, Cat. #2301, ScienCell Research Laboratories, Carlsbad, CA, USA) [11]. They were further maintained by changing the medium every other day or subjected to 3D spheroid cultures, as described in our previous study [11]. In brief, 2D cultured HconF cells were washed with phosphate-buffered saline (PBS), detached by treatment with 0.05% Trypsin/EDTA, re-suspended in the Fibroblast Medium supplemented with 0.25% methylcellulose (Methocel A4M) at a level of approximately 20,000 HconF cells in 28 μL, and each well was subjected to hanging drop culture plates (# HDP1385, Sigma-Aldrich, St. Louis, MO, USA) (Day 0). On each subsequent day until Day 6, half of the medium (14 μL) was exchanged with fresh medium. During Days 1 through 6, TGF-β2 (5 ng/mL) and/or all-trans-retinoic acid (ATRA, 1 μM) was added to the medium. These TGF-β2 and ATRA concentrations were confirmed as the optimum conditions based on previous studies [9,12].

### 2.2. Analysis of Barrier Function of the HconF Cell Monolayer by TEER and FITC Dextran Permeability

In the absence and presence of ATRA, HconF monolayers that had been treated with TGF-β2 (5 ng/mL) and untreated HconF monolayers were placed in each well of the TEER plate (0.4 μm pore size and 12 mm diameter; Corning Transwell, Sigma-Aldrich) and were cultured as above. On Day 6, the TEER values were measured using an electrical resistance system (KANTO CHEMICAL CO. INC., Tokyo, Japan), and FITC-dextran permeability was evaluated by measuring the permeated fluorescence intensity through the membrane from the basal compartment to the apical compartment during a period of 60 min as described in our previous study [13].

### 2.3. Analysis of Real-Time Bio-Cellular Metabolic Functions

For the bio-cellular function of the 2D HconF cells, the oxygen consumption rate (OCR) and the extracellular acidification (ECAR) of 2D HconF cells were evaluated by a Seahorse XFe96 Bioanalyzer (Agilent Technologies, Santa Clara, CA, USA), as described in our previous studies [14,15]. Briefly, 20 × 10^3^ 2D HconF cells were placed in wells of a 96-well assay plate as follows: (1) non-treated control (NT), (2) treated with TGF-β2, (3) treated with ATRA, and (4) treated with TGF-β2 and ATRA. After replacing the culture medium with Seahorse XF DMEM assay medium (pH 7.4, Agilent Technologies, #103575-100) supplemented with 5.5 mM glucose, 2.0 mM glutamine, and 1.0 mM sodium pyruvate, the basal OCR and ECAR values were determined using a Seahorse XFe96 Bioanalyzer (San Francisco, CA, USA), and thereafter, the samples were further analyzed after supplementation with 2.0 μM oligomycin, 5.0 μM carbonyl cyanide p-trifluoromethoxyphenylhydrazone (FCCP), 1.0 μM rotenone and antimycin A, and 10 mM 2-deoxyglucose (2-DG). The OCR and ECAR values were normalized to the amount of protein per well.

### 2.4. Evaluation of the Size and Hardness of HconF Cell 3D Spheroids

The physical properties, size, and hardness of the HconF 3D spheroids were characterized as reported in our previous studies [16,17]. In Brief, the mean sizes of the 3D spheroids were measured using an inverted microscope (Nikon ECLIPSE TS2; Tokyo, Japan). Alternatively, for the hardness measurement, a single living 3D spheroid was placed on a 3 mm × 3 mm plate and compressed until 50% deformation was achieved during 20 s using a micro-compressor (MicroSquisher, CellScale, Waterloo, ON, Canada). The force required (μN) was determined, and force/displacement (μN/μm) was calculated.

### 2.5. Immunocytechemictry of HconF Cells

The immunocytochemistry of the 2D and 3D cultured HconF cells was evaluated as described in our previous studies [18,19]. In brief, individual cells were fixed in 4% paraformaldehyde in PBS overnight, blocked in 3% BSA in PBS for 3 h, and washed twice with PBS for 30 min. They were then sequentially treated with (1) 1:200 dilutions of primary antibodies; an anti-human COL1 (#600-401-103-0.1, ROCKLAND Antibodies & Assays, Limerick, PA, USA), COL4 (#600-401-106S, ROCKLAND Antibodies & Assays, Limerick, PA, USA), COL6 (#009-001-108, ROCKLAND Antibodies & Assays, Limerick, PA, USA), or FN (#A-11, Santa Cruz Biotechnology, Inc., Dallas, TX, USA) rabbit antibody at 4 °C overnight, (2) washed 3 times with PBS for 1 h each, (3) 1:1000 dilutions of a secondary antibody; a goat anti-rabbit IgG (488 nm, #A27034, Invitrogen, Waltham, MA, USA) with phalloidin (594 nm, 1:1000 dilutions, # 17466-45-4, Cayman Chemical, Ann Arbor, MI, USA) and DAPI (1:1000 dilutions, # D523-10, DOJINDO, Osaka, Japan) for 3 hrs, and (4) mounting ProLong Gold Antifade Mountant with a cover glass. Immunofluorescent labeling images were obtained by means of a Nikon A1 confocal microscope (Nikon, Melville, NY, USA) using a ×20 air objective with a resolution of 1024 × 1024 pixels.

### 2.6. Other Analytical Methods

As previously reported [18,19], total RNA was extracted from 2D or 3D HconF cells using an RNeasy mini kit (Qiagen, Valencia, CA, USA) and was then subjected to reverse transcription using the SuperScript IV kit (Invitrogen) as per the manufacturer’s instructions. Each respective gene expression was quantified by real-time PCR with the Universal Taqman Master mix using a StepOnePlus machine (Applied Biosystems/Thermo Fisher Scientific) using specific primers and probes (Supplementary Table S1). cDNA quantities were normalized to the expression of 36B4 (Rplp0) and are shown as fold-change relative to that for the control.

All statistical analyses were performed using Graph Pad Prism 8 (GraphPad Software, San Diego, CA, USA), as described in a recent report [18,19]. A significant difference of less than 0.05 between experimental groups by ANOVA followed by Tukey’s multiple comparison test was determined to be statistically significant.

## 3. Results

**Figure 1:**
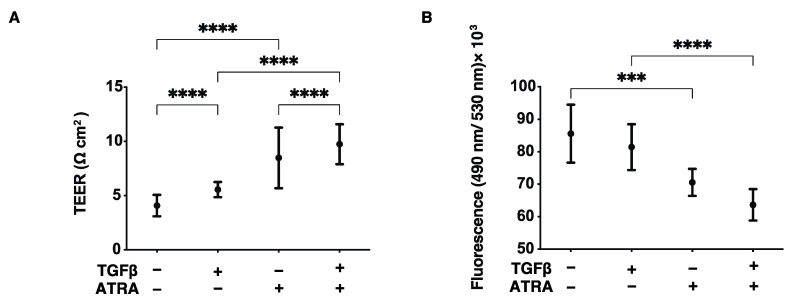
Effects of barrier functions of TGF-β2 untreated or treated HconF 2D monolayers. HconF cell 2D monolayers treated with TGF-β2 (TGFβ, 5 ng/mL) and untreated monolayers were subjected to analyses of TEER and FITC dextran permeability as their barrier functions in the absence or presence of 1 μM ATRA. Plots of the electric resistance (Ωcm^2^) by TEER and the absorbance of the permeated fluorescein are shown in panels (**A**,**B**), respectively. Experiments were repeated in triplicate (*n* = 5 each). All data are expressed as the mean ± the standard error of the mean (SEM). *** *p* < 0.005 and **** *p* < 0.001; ANOVA followed by a Tukey’s multiple comparison test.

**Figure 2:**
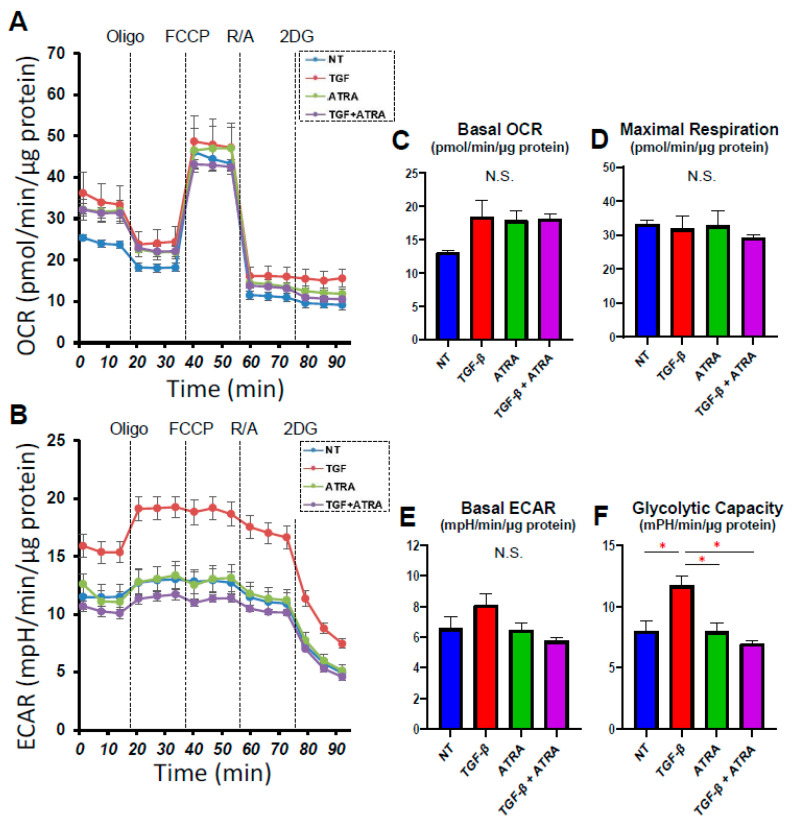
Effects of ATRA toward the mitochondrial glycolytic cellular functions of HconF 2D cells treated with TGF-β2 on untreated 2D HconF cells. Two-dimensional HconF cells untreated and treated with TGF-β2 (TGFβ, 5 ng/mL) were subjected to a seahorse real-time metabolic function analysis in the absence or presence of 1 μM ATRA. Panels (**A**,**B**): Basal OCR and ECAR were measured and then further measured by subsequent supplementation with a complex V inhibitor, oligomycin, a protonphore, FCCP, complex I/III inhibitors, rotenone/antimycin A, and a hexokinase inhibitor, 2DG. Panels (**C**,**E**): basal respiration of OCR and ECAR. Panel (**D**): maximal respiration calculated by subtraction of OCR with rotenone/antimycin A from OCR with FCCP. Panel (**F**): glycolytic capacity calculated by subtraction of ECAR with 2-DG from the baseline. Experiments were repeated in triplicate using fresh preparations (*n* = 5). Data are expressed as the mean ± the standard error of the mean (SEM). * *p* < 0.05; ANOVA followed by a Tukey’s multiple comparison test.

**Figure 3:**
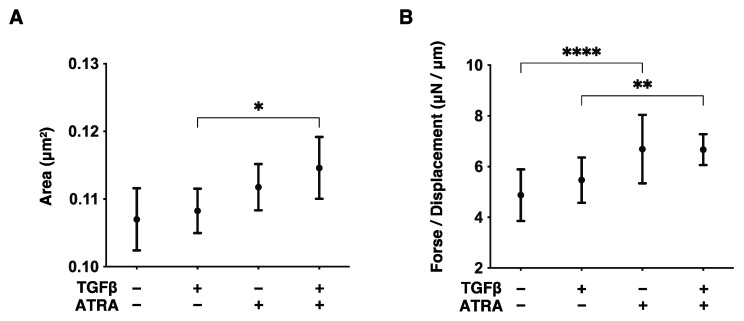
Effects of ATRA on the sizes and hardness of the TGF-β2 untreated or treated HconF 3D spheroids. Three-dimensional HconF spheroids untreated and treated with TGF-β2 (TGFβ, 5 ng/mL) were analyzed for their size and hardness measurements in the presence or absence of 1 μM ATRA. Mean sizes (μm^2^) and the force required to compress to the semidiameter (μN/μm) of a single 3D spheroid within 20 s were plotted in panels (**A**,**B**), respectively. Experiments were repeated in triplicate using fresh preparations (*n* = 16 spheroids each). Data are expressed as the mean ± standard error of the mean (SEM). * *p* < 0.05, ** *p* < 0.01 and **** *p* < 0.001; ANOVA followed by a Tukey’s multiple comparison test.

In order to investigate the effects of all-trans retinoic acid (ATRA) on conjunctival scarring, we employed our recently established in vitro model using 2D and 3D cultures of TGF-β2-treated HconF cells, which replicate the horizontal and vertical spreading of conjunctival scarring, respectively [11]. First, the ATRA-induced effects on the horizontal scaring direction of the conjunctiva were evaluated by trans-endothelial electron resistance (TEER) and FITC dextran permeability measurements of TGF-β2-treated and untreated HconF cell monolayers. As shown in Figure 1, upon exposure to a 5 ng/mL solution of TGF-β2, TEER values and FITC dextran permeability were found to be significantly increased or relatively decreased, respectively, as was observed in a previous study (Figure 1) [11]. Interestingly, 1 nM ATRA not only induced similar but more potent effects on TEER and FITC dextran permeability as TGF-β2 but also enhanced the TGF-β2-induced effects (Figure 1). To study this further, the effects of TGF-β2 and/or ATRA on real-time cellular metabolism was measured using a Seahorse Bioanalyzer. As shown in Figure 2, although neither TGF-β2 nor ATRA induced significant changes in the basal levels of OCR and ECAR, which represent mitochondrial respiration and glycolysis activities, respectively, glycolytic capacity was substantially increased by the mono-treatment of TGF-β2. These results indicate that the TGF-β2 and/or ATRA-induced effects on the TEER and FITC dextran permeability as above were independent of the mitochondrial respiration and glycolysis activities within HconF cells. Secondly, in terms of the effects of ATRA on the vertical spreading of conjunctival scarring, as shown in Figure 3, a mono-treatment of ATRA caused the formation of relatively larger and substantially stiffer 3D HconF spheroids, and those were further stimulated by TGF-β2. Therefore, these physical and functional analyses indicate that ATRA stimulated both TGF-β2-related and unrelated horizontal and vertical conjunctival scarring spreading with no significant effects on cellular metabolism.

**Figure 4:**
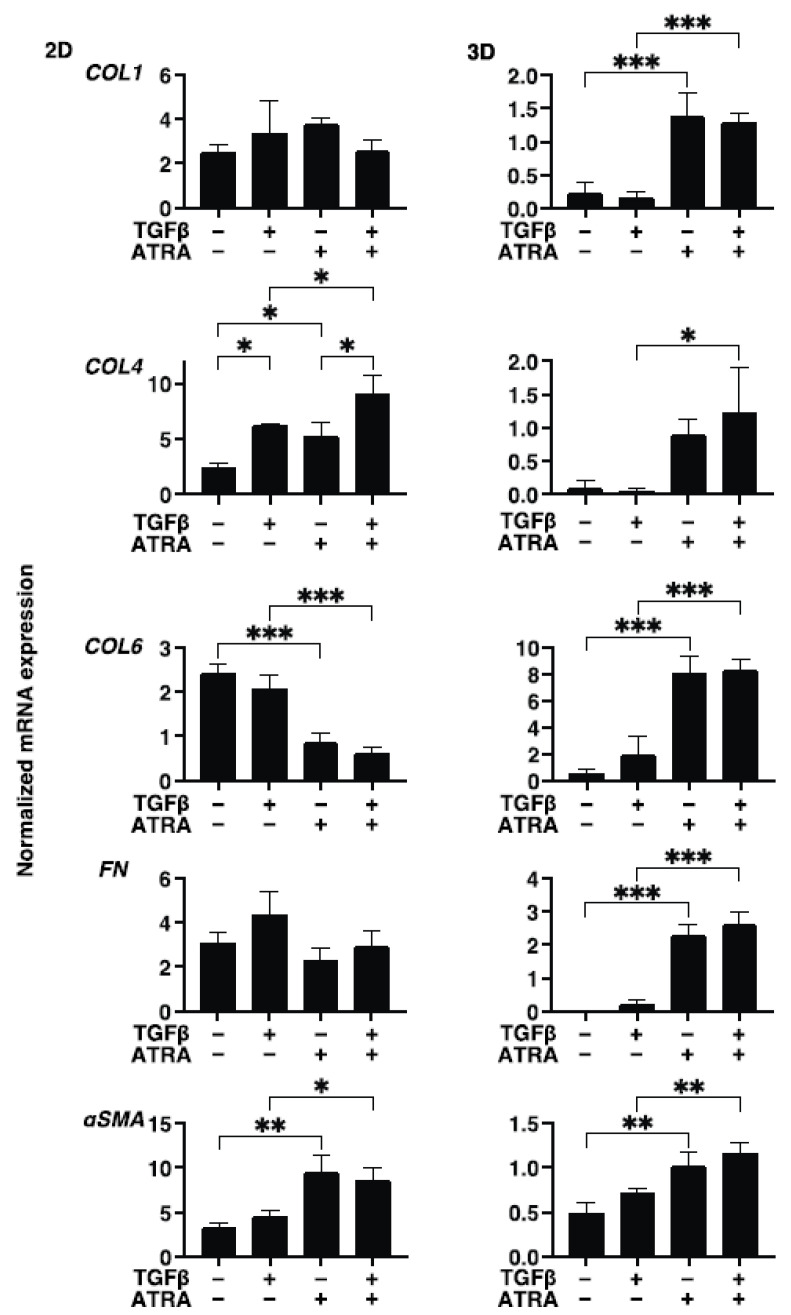
Effects of ATRA toward the gene expression of ECMs of the TGF-β2 untreated or treated HconF cells. Two-dimensional and three-dimensional HconF cells untreated and treated with TGF-β2 (TGFβ, 5 ng/mL) were subjected to qPCR analysis in the presence or absence of 1 μM ATRA and the mRNA expressions in ECMs, including *COL1*, *COL4*, *COL6*, *FN,* and *aSMA,* were evaluated. All experiments were performed in triplicate using 3 different confluent 6-well dishes (2D) or 15 freshly prepared 3D HconF spheroids (3D) in each experimental condition. Data are expressed as the mean ± standard error of the mean (SEM). * *p* < 0.05, ** *p* < 0.01 and *** *p* < 0.005; ANOVA followed by a Tukey’s multiple comparison test.

**Figure 5:**
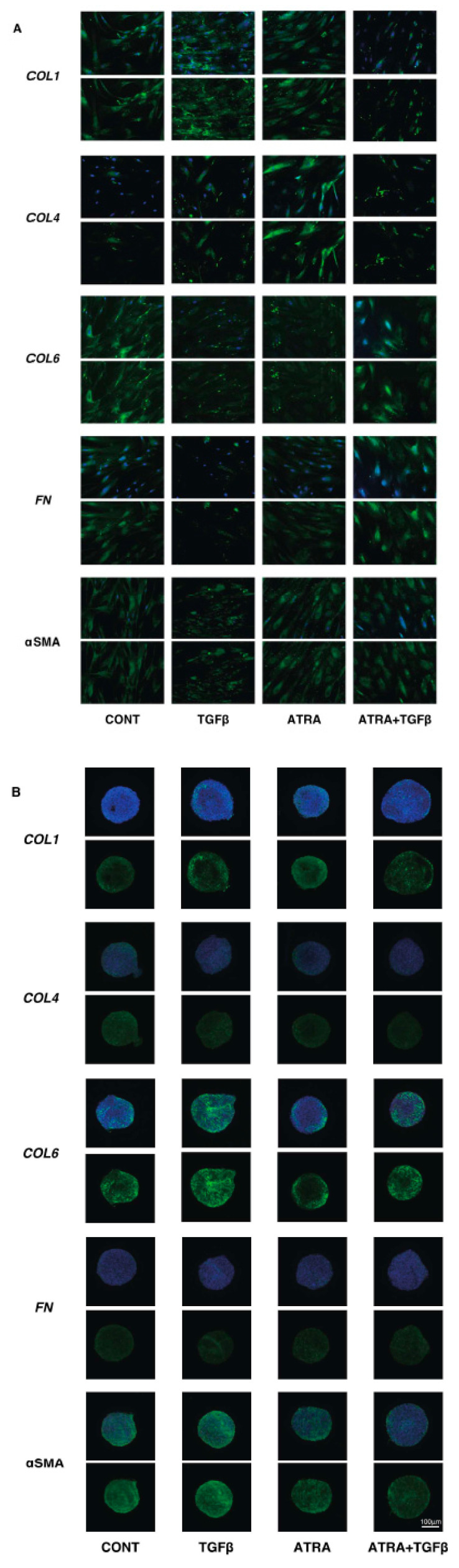
Effects of ATRA on the immunolabeling of ECM proteins of the TGF-β2 untreated or treated HconF cells. Two-dimensional and three-dimensional HconF cells untreated and treated with TGF-β2 (TGFβ, 5 ng/mL) were subjected to immunolabeling for *COL 1*, *COL 4*, *COL 6*, *FN,* and *aSMA* in the presence or absence of 1 μM ATRA. Experiments were repeated in duplicate (*n* = 5 in each). Representative images of ECM staining (lower) and the merged images with DAPI (upper) are shown in panels (**A**) (2D) and (**B**) (3D).

**Figure 6:**
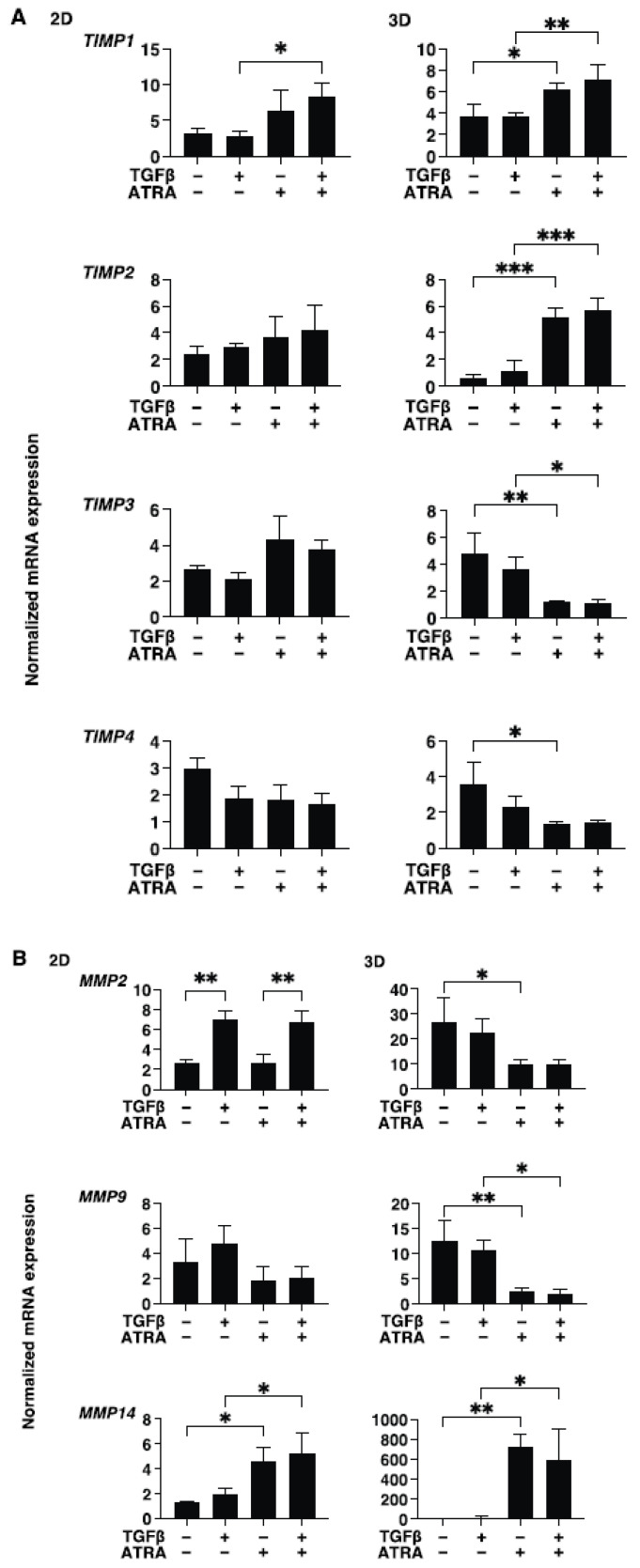
Effects of ATRA toward the gene expression of TIMPs and MMPs of the TGF-β2 treated and untreated HconF cells. Two-dimensional and three-dimensional HconF cells untreated and treated with TGF-β2 (TGFβ, 5 ng/mL) were subjected to qPCR analysis to evaluate the expression of mRNA in *TIMP1-4* (**A**) and *MMP2*, *9,* and *14* (**B**)*,* in the presence or absence of 1 μM ATRA. All experiments were performed in triplicate using 3 different confluent 6-well dishes (2D) or 15 freshly prepared 3D HconF spheroids (3D) in each experimental condition. Data are expressed as the mean ± standard error of the mean (SEM). * *p* < 0.05, ** *p* < 0.01 and *** *p* < 0.005; ANOVA followed by a Tukey’s multiple comparison test.

**Figure 7:**
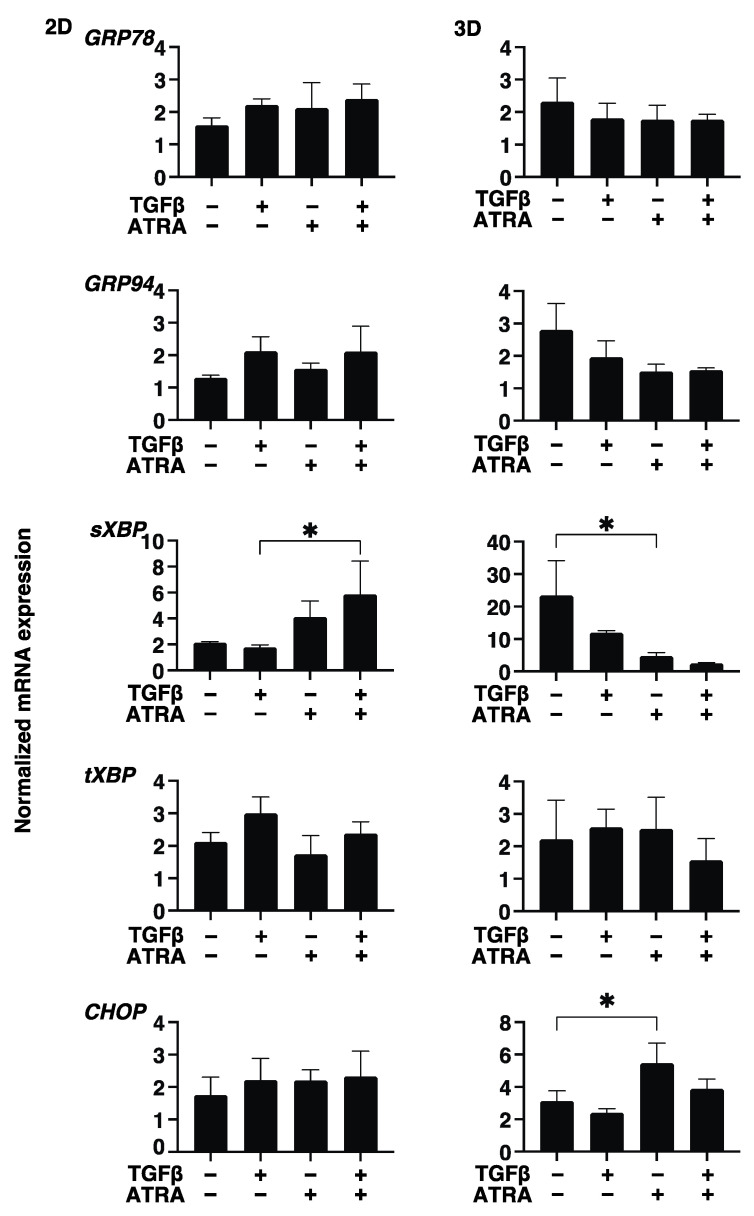
Effects of ATRA toward the gene expression of ER-stress related factors of the TGF-β2 untreated or treated HconF cells. Two-dimensional and three-dimensional HconF cells untreated and treated with TGF-β2 (TGFβ, 5 ng/mL) were subjected to qPCR analysis to evaluate the expression of mRNA of ER stress-related genes, the glucose regulator protein (GRP)78, GRP94, the X-box binding protein-1 (XBP1), spliced XBP1 (sXBP1), and CCAAT/enhancer-binding protein homologous protein (CHOP), in the presence or absence of 1 μM ATRA. All experiments were performed in triplicate using 3 different confluent 6-well dishes (2D) or 15 freshly prepared 3D HconF spheroids (3D) in each experimental condition. Data are expressed as the mean ± standard error of the mean (SEM). * *p* < 0.05; ANOVA followed by a Tukey’s multiple comparison test.

To study these issues further and the expressions of major ECM molecules, their modulators, including TIMP, MMP, and ER stress-related factors, were analyzed by qPCR and/or immunocytochemistry. Regarding the mRNA expressions of ECM molecules, TGF-β2 was found to only induce the upregulation of COL4 (2D) (Figure 4). In contrast, ATRA caused significant but different changes; that is, for 2D (upregulation: COL4 and αSMA, and downregulation: COL6) and 3D (significant upregulation: COL1, COL6, FN, and αSMA, relative upregulation: COL4) (Figure 4). Such TGF-β2 and or ATRA-induced changes were consistent with immunocytochemistry findings, except for COL6 and FN (3D) (Figure 5). Regarding this difference between mRNA expressions and the immunolabeling within the 3D spheroids, this was also reported in our previous study using cells from several sources [17,18,19,20,21]. As a possible reason for this, we speculate that immunolabeling may be representative of the expression of target molecules that are located on the surface of the 3D spheroids rather than the total expressions detected by qPCR analysis. Similar to these effects observed in ECM molecules, the gene expression of their modulators, TIMPs, MMPs, and ER-stress-related factors, were not significantly affected by TGF-β2, except for the upregulation of MMP2 (2D). In the case of 2D and 3D cultures, ATRA significantly modulated the expressions of these molecules but in different manners (Figure 6 and Figure 7). That is, in the case of 2D HconF cells, MMP 14 was upregulated, but in the case of 3D HconF spheroids, TIMP1 and 2, MMP14, and CHOP were upregulated, and TIMP3 and 4, MMP2 and 9, and sXBP were downregulated. Thus, these collective results suggest that ATRA exerts unfavorable effects on human conjunctival scarring itself by stimulating TGF-β2-induced effects, especially in the case of spreading in the vertical direction.

## 4. Discussion

It is known that vitamin A is required not only for the development of the eyes but also has several biological roles through an enzymatic derivative, retinoic acid (RA), which is produced in an autocrine or paracrine manner by binding to the nuclear retinoic acid receptors (RAR) α, β, and γ, and the retinoid X receptors (RXR) α, β, and γ [22]. It is known that RAR or RXR proteins are converted into biologically active forms by binding with ATRA and 9-cis-RA, or only 9-cis-RA [22]. Both RAR and RXR proteins, which form the functional unit of a RAR/RXR heterodimer, are expressed in the developing eye [23,24], and severe congenital eye defects have been reported in vitamin A deficient (VAD) and/or RA-deficient fetuses [25,26,27,28]. Alternatively, RA is also known to regulate the homeostasis of the blood–brain barrier (BBB) [29] as well as the blood–retinal barrier (BRB) [30] by the maintenance of tight junctions (TJs) in addition to the RA that is related to the normal development of the eyes. In fact, in a previous study, we reported that RAR alpha caused beneficial effects on vascular leakage in diabetic retinopathy (DR) by inhibiting the DR-induced deterioration of TJ [31]. These collective observations suggest that RA may also exert significant effects on both healthy and photogenic ocular surface barriers, including conjunctival tissues, as has been suggested in a previous review article [32]. In the current study, to elucidate possible unidentified RA-induced effects on fibrogenic changes in conjunctival tissues, we employed our recently developed in vitro models that replicate planer and subepithelial fibrogenesis using TGF-β2-treated or untreated 2D and 3D cultures of HconF cells and the following observations were obtained: ATRA caused (1) a significant increase and decrease in TEER values and FITC dextran permeability, respectively, of the 2D monolayers, (2) a substantial suppression of the TGF-β2-induced glycolytic capacity of 2D cells, (3) relative enlargement and significantly stiffer of the 3D spheroids, and (4) significant and different modulations of the mRNA expression of ECM proteins and their modulators between 2D and 3D cells. In addition, most of these ATRA-induced effects were additive to the TGF-β2-induced effects, except for glycolytic capacity. Therefore, based on these collective results, we conclude that ATRA may play a role in the deterioration associated with both planer and subepithelial conjunctival fibrosis and that this deterioration is independent of TGF-β2.

Consistent with this observation, an RA-induced increase in TEER values was also reported in intestinal epithelial cell monolayers [33] and capillary endothelial monolayers [31]. As possible underlying mechanisms for causing such an RA-induced TEER enhancement, both studies suggested that RA upregulated tight junction (TJ)-related molecules, such as claudin, occludin, and ZO-1, resulted in a mechanically stronger biological barrier. Indeed, such RA-induced upregulations of TJ-related molecules were reported in various non-cancerous [34] and cancerous epithelia. [35] However, since it was shown that a TJ complex is essentially not present within fibroblasts [36], an alternate mechanism is likely involved in the current observation of an ATRA-induced increase in TEER values. To support this possibility, such ATRA-induced changes in the TEER values of HconF cells (less than 10) were much less than those in the epithelial cells (80–150) [36]. Unfortunately, as of this writing, our knowledge of which mechanisms are involved is very limited. However, since our cellular metabolic analysis showed no significant changes in both OCR and ECAR, except that glycolytic capacity was inhibited in the presence of TGF-β2, we speculate that such unidentified mechanisms would require the minimum consumption of energy that may be enhanced by TGF-β2. In fact, in addition to the present observation that most of the effects of ATRA were enhanced by the presence of TGF-β2, as described above, RA signaling was associated with TGF signaling [5,37].

During the tissue remodeling processes, the metabolic balance between ECM proteins, i.e., degradation and synthesis, in addition to inter-cellular adhesion, is perturbed [38,39], and these mechanisms are also known to be affected by TGF-β2 [40] and ATRA [41,42] in several tissues. Among TGF-β-mediated signaling, it is well known that two different pathways, namely, smad-dependent [43] or smad-independent pathways, including the MAPK and PI3K/AKT pathways [44], are involved in fibroblast proliferation, migration, and ECM deposition. Furthermore, a previous study reported that ATRA suppresses TGF-β–induced contraction of the collagen gel sheet in human tenon fibroblasts by inhibiting MAPK, c-Jun, and SMAD signaling, suggesting that ATRA may beneficially inhibit conjunctival scarring [9,12]. However, in contrast, opposite effects of ATRA have been reported. That is, it has been reported that the administration of ATRA to corneal stromal keratinocytes induced an increase in the production of ECM components such as COL1 and reduced the production of MMP1, 3, and 9 [45]. Interestingly, it has been suggested that MMP9 plays a critical role in the wound healing processes by regulating re-epithelialization and cell migration [46,47], as indicated by the fact that an abnormal elevation of MMP9 is correlated with abnormal re-epithelialization [48]. Furthermore, the treatment of MCF-7 human breast cancer cells with ATRA, resulted in substantial inhibition of the expression and activity of the pro-MMP2 enzyme as well as a significant induction in TIMP2 protein expression [49]. Since the activity of MMPs in the extracellular space is specifically inhibited by TIMPs, it would be expected that the overexpression of TIMP2 would rationally inhibit the activity of MMP2 [50]. In addition, such retinoic acid-induced upregulation of TIMP2 expression was reported in the case of endothelial cells [51]. In the current study, the gene expressions of MMPs (2 and 9) of 3D HconF spheroids were also significantly downregulated, and, in turn, TIMP1 and 2 were substantially upregulated. Based upon these ATRA-induced changes in MMPs/TIMPs, our current observation of the significant upregulation of COL1 and other ECM proteins, especially in 3D HconF spheroids, provides reasonable support for the latter possibility that ATRA stimulates ECM production [45].

In recent decades, to replicate pathophysiological conditions closer to in vivo states, several 3D models of various tissues, including ocular tissues, have been developed [52,53,54]. Since the eye and periocular tissues are comprised of a large variety of heterogeneous tissues, most of which are spatially distributed within the 3D ocular cone area, 3D culture models would be expected to be more desirable for collecting more valid information in related research fields [52,53,54]. Quite recently, our group independently established in vitro 3D models using human orbital fibroblasts (HOFs), human corneal stromal fibroblasts (HCSFs) [16,19,21,55], human trabecular meshwork (HTM) cells [17,20,56,57,58], and human retinal pigment epithelium (HRPE) cells [59], in addition to HconF cells [11], and found that the biological natures of those 3D cell culture models were all quite different from those of their 2D culture cells. In the current study, such diversity between 2D and 3D cell cultures was also observed within the TGF-β2 treated and untreated HconF cells. However, in addition to the unidentified underlying mechanisms responsible for causing such diversity between 2D and 3D cultures, the findings indicated that TGF-β isoforms and retinoids, including ATRA, each exert complicated and multifunctional effects in development, physiology, and disease and also interact with each other in numerous biological settings [5]. In fact, retinoids both suppress and amplify TGF-β signaling dependent upon the tissue that is being considered, as well as in cellular and molecular settings [5]. Therefore, to develop a better understanding in terms of the ATRA-induced effects on conjunctiva and their scarring, as well as to elucidate unidentified issues that might be related to the effects on TEER and crosslinking with TGF-β2, additional studies using several genetically modified animals, RNA sequencing, and others will be required.

## Data Availability

The datasets used and/or analyzed during the current study are available from the corresponding author upon reasonable request.

## Supplementary Materials

The following supporting information can be downloaded at: https://www.mdpi.com/article/10.3390/bioengineering9090463/s1, Table S1: Sequences of primers used in qPCR.

## References

[1] Cordeiro M., Occleston N., Khaw P. (1997). New Concepts: Manipulation of the Wound-Healing Response. Dev. Ophthalmol..

[2] Cordeiro M.F., Chang L., Lim K.S., Daniels J.T., Pleass R.D., Siriwardena D., Khaw P.T. (2000). Modulating conjunctival wound healing. Eye.

[3] Saika S., Yamanaka O., Okada Y., Tanaka S.-I., Miyamoto T., Sumioka T., Kitano A., Shirai K., Ikeda K. (2009). TGFb in fibroproliferative diseases in tshe eye. Front. Biosci..

[4] Axel D.I., Frigge A., Dittmann J., Runge H., Spyridopoulos I., Riessen R., Viebahn R., Karsch K.R. (2001). All-trans retinoic acid regulates proliferation, migration, differentiation, and extracellular matrix turnover of human arterial smooth muscle cells. Cardiovasc. Res..

[5] Xu Q., Kopp J.B. (2012). Retinoid and TGF-β Families: Crosstalk in Development, Neoplasia, Immunity, and Tissue Repair. Semin. Nephrol..

[6] Na S.-Y., Kang B.Y., Chung S.W., Han S.-J., Ma X., Trinchieri G., Im S.-Y., Lee J.W., Kim T.S. (1999). Retinoids Inhibit Interleukin-12 Production in Macrophages through Physical Associations of Retinoid X Receptor and NFκB. J. Biol. Chem..

[7] Wen X., Li Y., Hu K., Dai C., Liu Y. (2005). Hepatocyte Growth Factor Receptor Signaling Mediates the Anti-Fibrotic Action of 9-cis-Retinoic Acid in Glomerular Mesangial Cells. Am. J. Pathol..

[8] Yang K.-L., Chang W.-T., Hung K.-C., Li E.I., Chuang C.-C. (2008). Inhibition of transforming growth factor-β-induced liver fibrosis by a retinoic acid derivative via the suppression of Col 1A2 promoter activity. Biochem. Biophys. Res. Commun..

[9] Liu Y., Kimura K., Orita T., Teranishi S., Suzuki K., Sonoda K.-H. (2014). Inhibition by All-Trans-Retinoic Acid of Transforming Growth Factor-β–Induced Collagen Gel Contraction Mediated by Human Tenon Fibroblasts. Investig. Opthalmology Vis. Sci..

[10] Liang L., Wang X., Zheng Y., Liu Y. (2019). All-trans-retinoic acid modulates TGF-β-induced apoptosis, proliferation, migration and extracellular matrix synthesis of conjunctival fibroblasts by inhibiting PI3K/AKT signaling. Mol. Med. Rep..

[11] Oouchi Y., Watanabe M., Ida Y., Ohguro H., Hikage F. (2021). Rosiglitasone and ROCK Inhibitors Modulate Fibrogenetic Changes in TGF-β2 Treated Human Conjunctival Fibroblasts (HconF) in Different Manners. Int. J. Mol. Sci..

[12] Liu Y., Kimura K., Orita T., Teranishi S., Suzuki K., Sonoda K.-H. (2015). All-trans-retinoic acid inhibition of transforming growth factor-β-induced collagen gel contraction mediated by human Tenon fibroblasts: Role of matrix metalloproteinases. Br. J. Ophthalmol..

[13] Kaneko Y., Ohta M., Inoue T., Mizuno K., Isobe T., Tanabe S., Tanihara H. (2016). Effects of K-115 (Ripasudil), a novel ROCK inhibitor, on trabecular meshwork and Schlemm’s canal endothelial cells. Sci. Rep..

[14] Sato T., Chang H.-C., Bayeva M., Shapiro J.S., Ramos-Alonso L., Kouzu H., Jiang X., Liu T., Yar S., Sawicki K.T. (2018). mRNA-binding protein tristetraprolin is essential for cardiac response to iron deficiency by regulating mitochondrial function. Proc. Natl. Acad. Sci. USA.

[15] Sato T., Ichise N., Kobayashi T., Fusagawa H., Yamazaki H., Kudo T., Tohse N. (2022). Enhanced glucose metabolism through activation of HIF-1α covers the energy demand in a rat embryonic heart primordium after heartbeat initiation. Sci. Rep..

[16] Hikage F., Atkins S., Kahana A., Smith T.J., Chun T.-H. (2019). HIF2A–LOX Pathway Promotes Fibrotic Tissue Remodeling in Thyroid-Associated Orbitopathy. Endocrinology.

[17] Ota C., Ida Y., Ohguro H., Hikage F. (2020). ROCK inhibitors beneficially alter the spatial configuration of TGFβ2-treated 3D organoids from a human trabecular meshwork (HTM). Sci. Rep..

[18] Ida Y., Hikage F., Itoh K., Ida H., Ohguro H. (2020). Prostaglandin F2α agonist-induced suppression of 3T3-L1 cell adipogenesis affects spatial formation of extra-cellular matrix. Sci. Rep..

[19] Itoh K., Hikage F., Ida Y., Ohguro H. (2020). Prostaglandin F2α Agonists Negatively Modulate the Size of 3D Organoids from Primary Human Orbital Fibroblasts. Investig. Opthalmology Vis. Sci..

[20] Watanabe M., Ida Y., Ohguro H., Ota C., Hikage F. (2021). Establishment of appropriate glaucoma models using dexamethasone or TGFβ2 treated three-dimension (3D) cultured human trabecular meshwork (HTM) cells. Sci. Rep..

[21] Itoh K., Ida Y., Ohguro H., Hikage F. (2021). Prostaglandin F2α agonists induced enhancement in collagen1 expression is involved in the pathogenesis of the deepening of upper eyelid sulcus. Sci. Rep..

[22] Chambon P. (1996). A decade of molecular biology of retinoic acid receptors. FASEB J..

[23] Ghyselinck N.B., Dupe V., Dierich A., Messaddeq N., Garnier J.M., Rochette-Egly C., Chambon P., Mark M. (1997). Role of the retinoic acid receptor beta (RARbeta) during mouse development. Int. J. Dev. Biol..

[24] Mori M., Ghyselinck N.B., Chambon P., Mark M. (2001). Systematic immunolocalization of retinoid receptors in developing and adult mouse eyes. Investig. Ophthalmol. Vis. Sci..

[25] Warkany J., Schraffenberger E. (1946). Congenital malformations induced in rats by maternal vitamin a deficiency. Arch. Ophthalmol..

[26] Wilson J.G., Roth C.B., Warkany J. (1953). An analysis of the syndrome of malformations induced by maternal vitamin a deficiency. Effects of restoration of vitamin a at various times during gestation. Am. J. Anat..

[27] Dickman E., Thaller C., Smith S. (1997). Temporally-regulated retinoic acid depletion produces specific neural crest, ocular and nervous system defects. Development.

[28] Dupé V., Matt N., Garnier J.-M., Chambon P., Mark M., Ghyselinck N.B. (2003). A newborn lethal defect due to inactivation of retinaldehyde dehydrogenase type 3 is prevented by maternal retinoic acid treatment. Proc. Natl. Acad. Sci. USA.

[29] Mizee M., Wooldrik D., Lakeman K.A.M., Hof B.V.H., Drexhage J.A.R., Geerts D., Bugiani M., Aronica E., Mebius R.E., Prat A. (2013). Retinoic Acid Induces Blood-Brain Barrier Development. J. Neurosci..

[30] Pollock L.M., Xie J., Bell B.A., Anand-Apte B. (2018). Retinoic acid signaling is essential for maintenance of the blood-retinal barrier. FASEB J..

[31] Nishikiori N., Osanai M., Chiba H., Kojima T., Mitamura Y., Ohguro H., Sawada N. (2007). Glial Cell–Derived Cytokines Attenuate the Breakdown of Vascular Integrity in Diabetic Retinopathy. Diabetes.

[32] Samarawickrama C., Chew S., Watson S. (2015). Retinoic acid and the ocular surface. Surv. Ophthalmol..

[33] Xiao S., Li Q., Hu K., He Y., Ai Q., Hu L., Yu J. (2018). Vitamin A and Retinoic Acid Exhibit Protective Effects on Necrotizing Enterocolitis by Regulating Intestinal Flora and Enhancing the Intestinal Epithelial Barrier. Arch. Med. Res..

[34] Osanai M., Nishikiori N., Murata M., Chiba H., Kojima T., Sawada N. (2007). Cellular Retinoic Acid Bioavailability Determines Epithelial Integrity: Role of Retinoic Acid Receptor α Agonists in Colitis. Mol. Pharmacol..

[35] Osanai M., Murata M., Nishikiori N., Chiba H., Kojima T., Sawada N. (2007). Occludin-mediated premature senescence is a fail-safe mechanism against tumorigenesis in breast carcinoma cells. Cancer Sci..

[36] Shi Y., Li R., Yang J., Li X. (2020). No tight junctions in tight junction protein-1 expressing HeLa and fibroblast cells. Int. J. Physiol. Pathophysiol. Pharmacol..

[37] Frenz D.A., Liu W., Cvekl A., Xie Q., Wassef L., Quadro L., Niederreither K., Maconochie M., Shanske A. (2010). Retinoid signaling in inner ear development: A “Goldilocks” phenomenon. Am. J. Med. Genet. Part A.

[38] Pelham R.J., Wang Y.-L. (1997). Cell locomotion and focal adhesions are regulated by substrate flexibility. Proc. Natl. Acad. Sci. USA.

[39] Wolf K., Lindert M.T., Krause M., Alexander S., Riet J.T., Willis A.L., Hoffman R.M., Figdor C.G., Weiss S.J., Friedl P. (2013). Physical limits of cell migration: Control by ECM space and nuclear deformation and tuning by proteolysis and traction force. J. Cell Biol..

[40] Cong M., Iwaisako K., Jiang C., Kisseleva T. (2012). Cell Signals Influencing Hepatic Fibrosis. Int. J. Hepatol..

[41] Fisher G.J., Datta S.C., Talwar H.S., Wang Z.-Q., Varani J., Kang S., Voorhees J.J. (1996). Molecular basis of sun-induced premature skin ageing and retinoid antagonism. Nature.

[42] Griffiths C., Russman A.N., Majmudar G., Singer R.S., Hamilton T.A., Voorhees J.J. (1993). Restoration of Collagen Formation in Photodamaged Human Skin by Tretinoin (Retinoic Acid). N. Engl. J. Med..

[43] Wen J., Lin X., Gao W., Qu B., Ling Y., Liu R., Yu M. (2019). MEK inhibition prevents TGF-β1-induced myofibroblast transdifferentiation in human tenon fibroblasts. Mol. Med. Rep..

[44] Zhang Y.E. (2009). Non-Smad pathways in TGF-β signaling. Cell Res..

[45] Gouveia R.M., Connon C.J. (2013). The Effects of Retinoic Acid on Human Corneal Stromal Keratocytes Cultured In Vitro Under Serum-Free Conditions. Investig. Opthalmology Vis. Sci..

[46] Kim E.C., Kim T.K., Park S.H., Kim M.S. (2012). The wound healing effects of vitamin A eye drops after a corneal alkali burn in rats. Acta Ophthalmol..

[47] Gordon G.M., Ledee D.R., Feuer W.J., Fini M.E. (2009). Cytokines and signaling pathways regulating matrix metalloproteinase-9 (MMP-9) expression in corneal epithelial cells. J. Cell. Physiol..

[48] Rayment E., Upton Z., Shooter G. (2008). Increased matrix metalloproteinase-9 (MMP-9) activity observed in chronic wound fluid is related to the clinical severity of the ulcer. Br. J. Dermatol..

[49] Dutta A., Sen T., Banerji A., Das S., Chatterjee A. (2009). Studies on Multifunctional Effect of All-Trans Retinoic Acid (ATRA) on Matrix Metalloproteinase-2 (MMP-2) and Its Regulatory Molecules in Human Breast Cancer Cells (MCF-7). J. Oncol..

[50] Westermarck J., Kähäri V.-M. (1999). Regulation of matrix metalloproteinase expression in tumor invasion. FASEB J..

[51] Braunhut S., Moses M. (1994). Retinoids modulate endothelial cell production of matrix-degrading proteases and tissue inhibitors of metalloproteinases (TIMP). J. Biol. Chem..

[52] García-Posadas L., Diebold Y. (2020). Three-Dimensional Human Cell Culture Models to Study the Pathophysiology of the Anterior Eye. Pharmaceutics.

[53] Aydin M., Dietrich J., Witt J., Finkbeiner M.S.C., Park J.J.-H., Wirth S., Engeland C.E., Paulsen F., Ehrhardt A. (2021). The Communication between Ocular Surface and Nasal Epithelia in 3D Cell Culture Technology for Translational Research: A Narrative Review. Int. J. Mol. Sci..

[54] Fiorentzis M., Katopodis P., Kalirai H., Seitz B., Viestenz A., Coupland S.E. (2019). Conjunctival melanoma and electrochemotherapy: Preliminary results using 2D and 3D cell culture models in vitro. Acta Ophthalmol..

[55] Ichioka H., Ida Y., Watanabe M., Ohguro H., Hikage F. (2021). Prostaglandin F2α and EP2 agonists, and a ROCK inhibitor modulate the formation of 3D organoids of Grave’s orbitopathy related human orbital fibroblasts. Exp. Eye Res..

[56] Watanabe M., Ida Y., Ohguro H., Ota C., Hikage F. (2021). Diverse effects of pan-ROCK and ROCK2 inhibitors on 2 D and 3D cultured human trabecular meshwork (HTM) cells treated with TGFβ2. Sci. Rep..

[57] Watanabe M., Ida Y., Furuhashi M., Tsugeno Y., Ohguro H., Hikage F. (2021). Screening of the Drug-Induced Effects of Prostaglandin EP2 and FP Agonists on 3D Cultures of Dexamethasone-Treated Human Trabecular Meshwork Cells. Biomedicines.

[58] Watanabe M., Sato T., Tsugeno Y., Umetsu A., Suzuki S., Furuhashi M., Ida Y., Hikage F., Ohguro H. (2022). Human Trabecular Meshwork (HTM) Cells Treated with TGF-β2 or Dexamethasone Respond to Compression Stress in Different Manners. Biomedicines.

[59] Suzuki S., Sato T., Watanabe M., Higashide M., Tsugeno Y., Umetsu A., Furuhashi M., Ida Y., Hikage F., Ohguro H. (2022). Hypoxia Differently Affects TGF-β2-Induced Epithelial Mesenchymal Transitions in the 2D and 3D Culture of the Human Retinal Pigment Epithelium Cells. Int. J. Mol. Sci..

