# Supplementary material for: All Trans-Retinoic Acids Facilitate the Remodeling of 2D and 3D Cultured Human Conjunctival Fibroblasts
Source: Bioengineering (Basel). 2022 Sep 11;9(9):463. doi: 10.3390/bioengineering9090463 (PMC9495389; doi:10.3390/bioengineering9090463)
Supplement: Supplementary file 1 [file bioengineering-09-00463-s001.zip › bioengineering-1832681-supplementary.pdf]

Table S1. Sequences of primers used in qPCR.

		Sequence	Exon Location	RefSeq Number
human RPLP0	Probe	5'-/56-FAM/CCCTGTCTT/ZEN/CCCTGGG-CATCAC/3IABkFQ/-3'	2-3	NM_001002
	Primer2	5'-TCGTCTTTAAACCCTGCGTG-3'		
	Primer1	5'-TGTCTGCTCCCACAATGAAAC-3'		
human COL1A1	Probe	5'-/56-FAM/TCGAGGGCC/ZEN/AAGACGAAGA-CATC/3IABkFQ/-3'	1-2	NM_000088
	Primer2	5'-GACATGTTCAGCTTTGTGGAC-3'		
	Primer1	5'-TTCTGTACGCAGGTGATTGG-3'		
human COL4A1	Probe	5′-/56-FAM/TCATACAGA/ZEN/CTTGG-CAGCGGCT/3IABkFQ/-3′	51-52	NM_001845
	Primer2	5′-AGAGAGGAGCGAGATGTTCA-3′		
	Primer1	5′-TGAGTCAGGCTTCATTATGTTCT-3′		
human COL6A1	Primer2	5'-CCTCGTGGACAAAGTCAAGT-3'	2-3	NM_001848
	Primer1	5'-GTGAGGCCTTGGATGATCTC-3'		
human FN1	Primer2	5'-CGTCCTAAAGACTCCATGATCTG-3'	3-4	NM_212482
	Primer1	5'-ACCAATCTTGTAGGACTGACC-3'		
human αSMA	Probe	5'-/56-FAM/AGACCCTGT/ZEN/TCCAGCCATCCTTC/3IAB-kFQ/-3'	8-9	NM_001613
	Primer2	5'-AGAGTTACGAGTTGCCTGATG-3'		
	Primer1	5'-CTGTTGTAGGTGGTTTCATGGA-3'		
human TIMP1	Probe	5′-/56-FAM/TCAACCAGA/ZEN/CCACCTTATAC-CAGCG/3IABkFQ/-3′	2-4	NM_003254
	Primer2	5′-CCTTCTGCAATTCCGACCT-3′		
	Primer1	5′-GCTTGGAACCCTTTATACATCTTG-3′		
human TIMP2	Probe	5′-/56-FAM/TCTCATTGC/ZEN/AG-GAAAGGCCGAGG/3IABkFQ/-3′	3-4	NM_003255
	Primer2	5′-GACGTTGGAGGAAAGAAGGA-3′		
	Primer1	5′-TGTGGTTCAGGCTCTTCTTC-3′		
human TIMP3	Probe	5′-/56-FAM/CCTCCTTTA/ZEN/CCAGCTTCTTCCCCAC/3IAB-kFQ/-3′	1-3	NM_000362
	Primer2	5′-CCTTCTGCAACTCCGACATC-3′		
	Primer1	5′-CGGTACATCTTCATCTGCTTGA-3′		
human TIMP4	Probe	5′-/56-FAM/ACTGAGGAC/ZEN/CTGACCAGTCAA-GAGA/3IABkFQ/-3′	3-4	NM_003256
	Primer2	5′-GGTTTGAGAAAGTCAAGGATGTTC-3′		
	Primer1	5′-GTTGCACAGATGGATGAAGAC-3′		
human MMP2	Primer2	5′-TCCACCACCTACAACTTTGAG-3′	6-7	NM_004530
	Primer1	5′-GTGCAGCTGTCATAGGATGT-3′		
human MMP9	Primer2	5′-ACATCGTCATCCAGTTTGGTG-3′	3-4	NM_004994
	Primer1	5′-CGTCGAAATGGGCGTCT-3′		
human MMP14	Primer2	5′-TTCGCCGACTAAGCAGAAG-3′	1-1	NM_004995
	Primer1	5′-CTTGAATTCCTAGACCGCTGT-3′		
human GRP78	Forward	5'-CATCACGCCGTCCTATGTCG-3'		NM_005347
	Reverse	5'-CGTCAAAGACCGTGTTCTCG-3'		
human GRP94	Forward	5'-CTGGGACTGGGAACTTATGAATG-3'		NM_003299
	Reverse	5'-TCCATATTCGTCAAACAGACCAC-3'		

human sXBP	Forward	5'-GGTCTGCTGAGTCCGCAGCAGG-3'	AB076384
	Reverse	5'-GGGCTTGGTATATATGTGG-3'	
human tXBP	Forward	5'-AGTAGCAGCTCAGACTGCCA-3'	NM_005080
	Reverse	5'-CCTGGTTCTCAACTACAAGGC-3'	
human CHOP	Forward	5'-GGAGAACCAGGAAACGGAAAC-3'	NM_004083
	Reverse	5'-TCTCCTTCATGCGCTGCTTT-3'	
